# Impact of Metformin Use on Liver Function Tests in Diabetic Patients With Suspected Non-Alcoholic Fatty Liver Disease (NAFLD): A Cross-Sectional Analysis

**DOI:** 10.7759/cureus.90635

**Published:** 2025-08-21

**Authors:** Adeel Asghar, Amina Azam, Muhammad Mustafa Imran, Muhammad Rohail Tariq, Muhammad Adil Sohail, Zainab Tariq, Umer Mushtaq, Fajar Khalid

**Affiliations:** 1 Internal Medicine, Baba Bulleh Shah Hospital, Kasur, PAK; 2 General Medicine, Gujranwala Medical College Teaching Hospital, Gujranwala, PAK; 3 Medicine, King Edward Medical University, Lahore, PAK; 4 Internal Medicine, King Edward Medical University, Lahore, PAK; 5 Emergency Medicine, Hillingdon Hospital, London, GBR; 6 Geriatric Medicine, Ealing Hospital, London, GBR; 7 General Medicine, Jinnah Hospital, Lahore, PAK

**Keywords:** human pathophysiology, mechanism, metformin, nonalcoholic fatty liver disease (nafld), patients

## Abstract

Background: Non-alcoholic fatty liver disease (NAFLD) is highly prevalent among patients with type 2 diabetes mellitus (T2DM) and is often associated with elevated liver enzymes.

Objective: To assess the impact of metformin use on liver function tests in diabetic patients with clinically suspected NAFLD.

Methods: This was a cross-sectional analytical study conducted at Jinnah Hospital, Lahore, Pakistan, from July 2024 to December 2024. A total of 185 adult patients were enrolled in the study. Non-probability consecutive sampling was used to recruit participants who met the inclusion criteria. Demographic data, including age, gender, body mass index (BMI), and duration of diabetes, were recorded. Clinical records were reviewed to document metformin use (yes/no), dosage, and duration.

Results: Out of 185 patients, 115 (62.2%) were metformin users and 70 (37.8%) were non-users. Metformin users had significantly lower mean alanine aminotransferase (ALT) (41.3 ± 14.6 IU/L vs. 54.7 ± 18.2 IU/L; p < 0.001) and aspartate aminotransferase (AST) levels (35.2 ± 12.4 IU/L vs. 44.6 ± 15.7 IU/L; p = 0.002) compared to non-users. No significant differences were observed in alkaline phosphatase (ALP) and gamma-glutamyl transferase (GGT) levels. Elevated ALT and AST were significantly less frequent in metformin users (p < 0.01). A weak inverse correlation was observed between metformin dose and ALT levels (r = -0.19, p = 0.043).

Conclusion: It is concluded that metformin use is associated with improved liver enzyme profiles in diabetic patients with suspected NAFLD. These findings suggest a potential hepatoprotective role for metformin, warranting further longitudinal studies to explore its impact on liver histology and long-term hepatic outcomes.

## Introduction

Non-alcoholic fatty liver disease (NAFLD) has become the most prevalent cause of chronic liver disease globally. It affects more than a quarter of the adult population, with an even higher burden among individuals with type 2 diabetes mellitus (T2DM). The prevalence of NAFLD can go as high as 7080 in the diabetic population, and half of them are unaware of the early stages of liver-damaging activities because it produces no symptoms [1]. NAFLD is the continuum of hepatic pathologies extending from simple steatosis and non-alcoholic steatohepatitis (NASH) to advanced fibrosis, cirrhosis, and hepatocellular carcinoma [2]. It is this silent but progressive characteristic of the disease that is a major threat, especially where the patient has a coexisting metabolic dysfunction [3]. The relationship between NAFLD and T2DM is complicated and highly interconnected through similar pathophysiological processes, which include, among others, oxidative stress, insulin resistance, chronic inflammation, and deranged lipid profile. Consequently, the treatments involving insulin resistance could also bring about positive effects on the overall liver health [4]. Metformin is one such agent, and it is a first-line oral antidiabetic agent that reduces hepatic glucose production, stimulates peripheral glucose uptake, and enhances insulin sensitivity. In addition to its widely documented efficacy with respect to glycemic control, metformin is also gaining interest in terms of its influence on the progression of NAFLD [5].

Metformin binds one enzyme, AMP-activated protein kinase (AMPK), which plays an important role in energy homeostasis and regulates lipid production and fatty acid oxidation through the liver. By this, metformin can decrease the hepatic contents of fatty substances, inflammation, and fibrosis. Improvement in liver enzymes and in metabolic parameters was observed in some studies in patients using metformin, whereas other studies reported less encouraging or a lack of evidence of improvement in histology [6]. Regardless of these contradictory results, this drug has continued to be used extensively in the clinic in patients with undiagnosed or suspected NAFLD without consideration being given as to its effect on the liver. Such biomarkers as liver function tests (LFTs) represent alanine aminotransferase (ALT), aspartate aminotransferase (AST), alkaline phosphatase (ALP), and gamma-glutamyl transferase (GGT), which are convenient, non-invasive indicators used to evaluate the hepatic functioning [7]. LFTs are the commonest abnormalities in diabetic patients that lead to the first indications of underlying NAFLD. Even though the LFTs are not always related to histological severity, it has become a handy consultation that is used during routine monitoring and during clinical decision-making, particularly in resource-limited areas where images or tests requiring biopsy are limited [8]. Diabetes and NAFLD epidemics are becoming an increasingly threatening problem in most low- and middle-income states, including Pakistan [9]. Nonetheless, NAFLD is underrecognized, and its possible hepatic complications tend to be ignored in the course of managing diabetes. The metabolic abnormalities are often treated in patients with only slightly deranged liver enzymes, even without any further details about the hepatic condition [10]. Considering how popular and even how prolonged the usage of metformin is within these populations, it is highly understandable why there is an urgent need to determine the overall effects that the drug has on the liver status. Assuming that metformin does have hepatoprotective properties, the drug might become an affordable, dual-purpose treatment in a population with a combined load of T2DM and liver disease [11].

Objective

To evaluate the association between metformin use and liver enzyme levels in type 2 diabetic patients with suspected NAFLD.

## Materials and methods

This was a cross-sectional analytical study conducted at Jinnah Hospital, Lahore, Pakistan, from July 2024 to December 2024. A total of 185 adult patients were enrolled in the study. Non-probability consecutive sampling was used to recruit participants who met the inclusion criteria. While this method allowed for practical and timely recruitment, it may introduce selection bias and limit the generalizability of the findings to broader populations, as it does not ensure random representation. The inclusion criteria for this study consisted of adult patients aged 30-70 years who were diagnosed with type 2 diabetes mellitus (T2DM). Participants were required to have either clinical or ultrasonographic suspicion of non-alcoholic fatty liver disease (NAFLD) and must have been on stable antidiabetic therapy for at least three months. Additionally, the availability of recent liver function tests, conducted within the past 30 days, was required for inclusion. Patients were excluded with a history of chronic liver diseases, including hepatitis B, hepatitis C, or autoimmune hepatitis. Individuals with alcohol consumption exceeding 20 g/day for females and 30 g/day for males were also excluded. Furthermore, patients using hepatotoxic medications other than metformin, pregnant or lactating women, and those with end-stage renal disease or decompensated heart failure were not included in the study.

Data collection procedure

Demographic data, including age, gender, BMI, and duration of diabetes, were recorded. In this study, “suspected NAFLD” was defined using both clinical and ultrasonographic criteria. Clinically, patients were considered to have suspected NAFLD if they had type 2 diabetes mellitus along with risk factors such as obesity (BMI ≥25 kg/m²), insulin resistance, or metabolic syndrome, and mildly elevated liver enzymes (ALT and/or AST above the normal range), in the absence of other known causes of liver disease. Clinical records were reviewed to document metformin use (yes/no), dosage, and duration. Liver function tests (ALT, AST, ALP, and GGT) were obtained from laboratory reports. Patients were then grouped into two cohorts based on metformin use: metformin users and non-users. 

Statistical analysis

Data were entered and analyzed using IBM Corp. Released 2020. IBM SPSS Statistics for Windows, Version 26. Armonk, NY: IBM Corp. Quantitative variables such as liver enzyme levels and age were presented as mean ± standard deviation (SD), while qualitative variables were presented as frequencies and percentages. An independent sample t-test was used to compare mean liver enzyme levels between the two groups, based on the distribution of data. A p-value of less than 0.05 was considered statistically significant.

## Results

Among 185 participants, baseline characteristics such as age, gender, BMI, and diabetes duration were comparable between metformin users and non-users (p > 0.05). However, liver enzymes were significantly lower in metformin users: ALT (41.3 ± 14.6 vs. 54.7 ± 18.2 IU/L, p < 0.001) and AST (35.2 ± 12.4 vs. 44.6 ± 15.7 IU/L, p = 0.002). ALP and GGT differences were not statistically significant (Table 1).

**Table 1:** Baseline characteristics of study participants (n = 185) The test statistics for continuous variables were calculated using an independent samples t-test. The test statistic for categorical variables was calculated using a chi-square (χ²) test. BMI: Body mass index, ALT: Alanine aminotransferase, AST: Aspartate aminotransferase, ALP: Alkaline phosphatase, GGT: Gamma-glutamyl transferase

Variable	Metformin Users (n = 115)	Non-Users (n = 70)	Test Statistic	p-value
Mean Age (years)	54.2 ± 9.6	55.1 ± 10.1	t = 0.69	0.48
Gender (Male)	61 (53.0%)	37 (52.9%)	χ² = 0.0001	0.99
BMI (kg/m²)	28.7 ± 3.4	29.1 ± 3.6	t = -0.87	0.39
Duration of Diabetes (years)	8.9 ± 4.1	9.4 ± 3.8	t = -0.94	0.34
ALT (IU/L)	41.3 ± 14.6	54.7 ± 18.2	t = -5.47	<0.001
AST (IU/L)	35.2 ± 12.4	44.6 ± 15.7	t = -3.18	0.002
ALP (IU/L)	108.5 ± 32.9	112.4 ± 36.2	t = -0.80	0.42
GGT (IU/L)	51.7 ± 19.8	55.9 ± 22.3	t = -1.26	0.21

Elevated ALT was observed in 29.6% of metformin users compared to 58.6% of non-users (p < 0.001). Similarly, elevated AST was present in 23.5% vs. 45.7%, respectively (p = 0.002). No significant differences were found in the prevalence of elevated ALP (p = 0.33) or GGT (p = 0.49), indicating metformin’s more specific effect on transaminases (Table 2).

**Table 2:** Liver enzyme abnormalities among study participants The test statistic for categorical variables was calculated using a chi-square (χ²) test. ALT: Alanine aminotransferase, AST: Aspartate aminotransferase, ALP: Alkaline phosphatase, GGT: Gamma-glutamyl transferase

Enzyme Abnormality	Metformin Users (n = 115)	Non-Users (n = 70)	Test Statistic	p-value
Elevated ALT (>45 IU/L)	34 (29.6%)	41 (58.6%)	χ² = 20.55	<0.001
Elevated AST (>40 IU/L)	27 (23.5%)	32 (45.7%)	χ² = 9.73	0.002
Elevated ALP (>120 IU/L)	21 (18.3%)	17 (24.3%)	χ² = 1.02	0.33
Elevated GGT (>60 IU/L)	26 (22.6%)	19 (27.1%)	χ² = 0.47	0.49

Duration of diabetes was positively correlated with ALT (r = 0.24, p = 0.001), AST (r = 0.18, p = 0.017), and GGT (r = 0.15, p = 0.034), but not significantly with ALP (r = 0.11, p = 0.09). These findings suggest that longer diabetes duration is associated with mild liver enzyme elevation, particularly in ALT (Table 3).

**Table 3:** Correlation of duration of diabetes with liver enzyme levels (n = 185) The test statistic used was Pearson correlation to assess the relationship between duration of diabetes and liver enzyme levels. ALT: Alanine aminotransferase, AST: Aspartate aminotransferase, ALP: Alkaline phosphatase, GGT: Gamma-glutamyl transferase

Parameter	Pearson Correlation (r)	p-value
ALT	0.24	0.001
AST	0.18	0.017
ALP	0.11	0.09
GGT	0.15	0.034

Mean ALT levels decreased with increasing metformin dose. Patients on a low dose (<1000 mg/day) had a mean ALT of 45.8 ± 13.2 IU/L, compared to 41.1 ± 15.1 in the moderate-dose group and 38.7 ± 12.8 in the high-dose group. The reduction was statistically significant for high-dose users (p = 0.043), suggesting a dose-dependent hepatoprotective effect (Table 4).

**Table 4:** Subgroup analysis of ALT levels by metformin dose ALT: Alanine aminotransferase Test used: One-way analysis of variance (ANOVA). Interpretation: Higher doses of metformin are associated with lower ALT levels.

Metformin Dose Group	n	Mean ALT (IU/L) ± SD	Test Statistic	p-value
Low dose (<1000 mg/day)	28	45.8 ± 13.2	F = 3.45	Reference
Moderate dose (1000–1500 mg/day)	49	41.1 ± 15.1	F = 1.80	0.18
High dose (≥1500 mg/day)	38	38.7 ± 12.8	F = 4.10	0.043*

## Discussion

This study evaluated the effect of metformin use on liver function tests (LFTs) in diabetic patients with suspected non-alcoholic fatty liver disease (NAFLD). The results indicate that the use of metformin is significantly linked to low serum levels of alanine aminotransferase (ALT) and aspartate aminotransferase (AST), which have been proven by general acceptance to be used as remote indicators of hepatocellular injury. The findings in this study contribute to the mounting suspicion that, in people with underlying metabolic liver illness, metformin, over and above its glucose-lowering effects, can bring down the risk of hepatopathy. Mean levels of ALT and AST were significantly lower in users of metformin than in non-users, which hints that they had lower rates of hepatic inflammation or damage [12]. This coincides with earlier findings asserting that metformin modulates AMP-activated protein kinase (AMPK), which subsequently inhibits the content of hepatic lipids, insulin sensitivity, and potentially liver inflammation. Also, the frequency rate of patients with high levels of ALT and AST was significantly decreased in the metformin regimen, which confirms the presumption of the potential role of such medication in the stabilization of liver biochemistry or even the enhancement of liver biochemistry in the NAFLD patients [13].

Visibly, there was no difference in the results of alkaline phosphatase (ALP) or gamma-glutamyl transferase (GGT) levels between the groups. This can be considered an indication that the effects of metformin are more specific to hepatocellular damage compared to the effects of cholestasis or biliary involvement, which are more indicative of the elevated ALP and GGT levels [14]. Various non-hepatic influences, such as bone metabolism, also affect them and may render the specificity rather dilute in the scenario of NAFLD alone. There was a strong reverse correlation between metformin dose and level of ALT [15]. The mean values of the ALT levels of patients who received a higher dose (1500 mg/day) were lower than those who received lower doses, indicating a possible dose-response effect. These observations, however, must be viewed with caution since there are many variables that may operate as confounders, like duration of therapy, adherence, and baseline condition of the liver. Still, these observations are in need of further investigations in future dose-ranging studies [16].

The long-term survival of diabetes was moderately and significantly related to the elevated intervals of liver enzyme levels, when ALT and AST were the most strongly correlated. This confirms the idea that chronic hyperglycemia and insulin resistance accumulate hepatic stress with time, and early therapeutic treatment (i.e., use of metformin) may be of greater benefit when initiated earlier in the disease process [17]. Although these are encouraging results, one must take into consideration that metformin is not yet approved as a treatment of NAFLD in the non-diabetic patient and that its benefit on histopathological benefit (e.g., fibrosis regression) is yet to be determined [18]. Moreover, normalization of liver enzymes is not historically reversible, and the actual effects of metformin on liver architecture would demand a radiological or histopathological ascertainment. However, the biochemical outcome changes observed in the study are still clinically relevant, particularly in resource-limited areas where both liver biopsies and elastography might be less readily available [19]. The study also appears to point out the usefulness of monitoring hepatic functionality in diabetes patients, especially those who are not on metformin or those with established disease [20]. Routine biochemical monitoring can give early alerts of hepatic involvement and help inform treatment choice. Disadvantages of this research are down to its study design, which is cross-sectional and therefore prevents the establishment of causality. Due to the use of LFTs in isolation without imaging or histological diagnosis of NAFLD, including the absence of imaging and histology that, in isolation, cannot be interpreted or classified as NAFLD, the use of LFTs can underestimate or mislabel the severity of the disease. Besides, the data on dietary intake, physical activity, and any other medications that might affect the liver enzymes remained uncontrolled. Despite these restrictions, the study gives real-life data that support the hepatic safety and potential utility of metformin in diabetic patients who are suspected of NAFLD. Although the results indicate substantive relationships between metformin and better liver enzyme status in diabetic patients with possible NAFLD, it must be noted that the analysis lacks adjustment of possible confounding factors like body mass index, glycemic control indicators (i.e., HbA1c), and the use of other anti-diabetic drugs and/orany hepatoprotective drug, as well as the comorbidities like dyslipidemia and hypertension. Such aspects may affect the liver enzyme concentration independent of each other and may partly justify the differences between the metformin users and non-users. When multivariate analysis or regression modeling is not used, the real sense of the independent impact of metformin on hepatic biomarkers is questionable. Future researchers ought to make statistical corrections to these covariates so as to better isolate the individual contribution of metformin to hepatic effects.

## Conclusions

It is concluded that metformin use is associated with lower ALT and AST levels in diabetic patients with suspected non-alcoholic fatty liver disease (NAFLD), suggesting a possible beneficial relationship. While these findings support a potential link between metformin use and improved liver enzyme profiles, they should be interpreted as associative rather than causal. The results offer valuable insights from a resource-limited setting; however, longitudinal studies with appropriate confounder adjustment are needed to determine whether metformin exerts a true hepatoprotective effect. The findings emphasize the importance of considering metformin not only for its well-established role in glycemic control but also for its potential positive impact on liver health. However, further longitudinal studies are necessary to confirm these results, explore the long-term effects of metformin on liver histology, and fully understand its role in managing liver complications in diabetic patients.

## References

[1] Pinyopornpanish K, Leerapun A, Pinyopornpanish K, Chattipakorn N (2021). Effects of metformin on hepatic steatosis in adults with nonalcoholic fatty liver disease and diabetes: insights from the cellular to patient levels. Gut Liver.

[2] Steiger A, VanderVeen NT, Kang EH, Weimer AK (2024). Resolution of metabolic dysfunction-associated steatohepatitis with estradiol in a transgender female: A case report. JPGN Rep.

[3] Doycheva I, Loomba R (2014). Effect of metformin on ballooning degeneration in nonalcoholic steatohepatitis (NASH): when to use metformin in nonalcoholic fatty liver disease (NAFLD). Adv Ther.

[4] Green CJ, Marjot T, Tomlinson JW, Hodson L (2019). Of mice and men: Is there a future for metformin in the treatment of hepatic steatosis?. Diabetes Obes Metab.

[5] Tang W, Xu Q, Hong T (2016). Comparative efficacy of anti-diabetic agents on nonalcoholic fatty liver disease in patients with type 2 diabetes mellitus: a systematic review and meta-analysis of randomized and non-randomized studies. Diabetes Metab Res Rev.

[6] Ahmad R, Haque M (2024). Metformin: beyond type 2 diabetes mellitus. Cureus.

[7] Zachou M, Flevari P, Nasiri-Ansari N (2024). The role of anti-diabetic drugs in NAFLD. Have we found the Holy Grail? A narrative review. Eur J Clin Pharmacol.

[8] Chui ZS, Xue Y, Xu A (2024). Hormone-based pharmacotherapy for metabolic dysfunction-associated fatty liver disease. Med Rev (2021).

[9] Milošević N, Milanović M, Medić Stojanoska M (2025). Triterpenoids from Chios Mastiha resin against MASLD-a molecular docking survey. Curr Issues Mol Biol.

[10] Huang KH, Lee CH, Cheng YD (2022). Correlation between long-term use of metformin and incidence of NAFLD among patients with type 2 diabetes mellitus: A real-world cohort study. Front Endocrinol (Lausanne).

[11] Tomah S, Alkhouri N, Hamdy O (2020). Nonalcoholic fatty liver disease and type 2 diabetes: where do Diabetologists stand?. Clin Diabetes Endocrinol.

[12] Ciardullo S, Vergani M, Perseghin G (2023). Nonalcoholic fatty liver disease in patients with type 2 diabetes: screening, diagnosis, and treatment. J Clin Med.

[13] Wong VWS, Vergniol J (2010). Diagnosis of fibrosis and cirrhosis using liver stiffness measurement in nonalcoholic fatty liver disease. Hepatology.

[14] Siddiqui MS, Yamada G, Vuppalanchi R (2019). Diagnostic accuracy of noninvasive fibrosis models to detect change in fibrosis stage. Clin Gastroenterol Hepatol.

[15] European Association for Study of Liver (2015). EASL-ALEH Clinical Practice Guidelines: Non-invasive tests for evaluation of liver disease severity and prognosis. J Hepatol.

[16] Gastaldelli A, Cusi K, Fernández Landó L (2022). Effect of tirzepatide versus insulin degludec on liver fat content and abdominal adipose tissue in people with type 2 diabetes (SURPASS-3 MRI): a substudy of the randomised, open-label, parallel-group, phase 3 SURPASS-3 trial. Lancet Diabetes Endocrinol.

[17] Venkatesh SK, Ehman RL (2014). Magnetic resonance elastography of liver. Magn Reson Imaging Clin N Am.

[18] ElSayed NA, Aleppo G, Aroda VR (2023). Addendum. 4. Comprehensive medical evaluation and assessment of comorbidities: standards of care in diabetes-2023. Diabetes Care.

[19] Mantovani A, Byrne CD, Scorletti E (2020). Efficacy and safety of anti-hyperglycaemic drugs in patients with non-alcoholic fatty liver disease with or without diabetes: An updated systematic review of randomized controlled trials. Diabetes Metab.

[20] Newsome PN, Buchholtz K, Cusi K (2021). A placebo-controlled trial of subcutaneous semaglutide in nonalcoholic steatohepatitis. N Engl J Med.

